# Genomic and Pathogenic Characterization of an Extensively Drug‐Resistant Avian Pathogenic *Escherichia coli* Strain

**DOI:** 10.1155/tbed/4233989

**Published:** 2026-05-14

**Authors:** Weishi Ni, Lijuan Chen, Hao Chen, Kejing Wang, Lumin Yu, Xinglin Zhang, Peikun Wang

**Affiliations:** ^1^ College of Agriculture and Forestry, Linyi University, Linyi, 276000, Shandong, China, lyu.edu.cn

**Keywords:** avian pathogenic *Escherichia coli*, extensively drug-resistant, genome analysis, pathogenicity, plasmid

## Abstract

Extensively drug‐resistant (XDR) avian pathogenic *Escherichia coli* (APEC) poses a global threat to poultry and public health. In this study, we isolated an APEC strain, SDLYRA415, from broilers in Shandong, China, during an outbreak with ~20% mortality. To investigate the genomic characteristics, resistance mechanisms, and pathogenicity of SDLYRA415, drug susceptibility was tested by disk diffusion and minimum inhibitory concentration (MIC); whole‐genome sequencing analyzed genomic features and resistance genes; conjugation assays evaluated resistance transfer; animal experiments (BALB/c mice, chicks) assessed pathogenicity. Drug susceptibility test results showed that SDLYRA415 was resistant to 32 antibiotics, showed intermediate resistance to polymyxin B and amikacin, and was only susceptible to tigecycline. Whole‐genome sequencing showed SDLYRA415 had a 5.51 Mb genome, including one chromosome and four plasmids. Phylotyping, multilocus sequence typing (MLST), and serotyping classified SDLYRA415 as phylogroup A, ST48, and O6: H16. Plasmids 1, 2, and 4 belonged to IncHI2/IncHI2A/IncN, IncFIB(AP001918)/IncFIC(FII), and p0111 groups, respectively, while plasmid 3 was a novel type. A total of 78 antibiotic resistance genes (ARGs) were detected, including *bla*_CTX-M−55_, *MCR-1*, *bla*_CTX-M−65_, *NDM-5*, etc. Conjugation assays showed that SDLYRA415 transferred polymyxin B resistance to EC600 at 6.5 × 10^−7^efficiency, and EC600 recipient strains levels of resistance showed significant increases. Pathogenicity tests showed that SDLYRA415 caused 60% mortality in both BALB/c mice and chicks, demonstrating its potential to cause severe disease in mammalian models and underscoring its zoonotic threat. This study offers crucial insights into drug resistance and pathogenic patterns of XDR‐APEC, aiding in the development of targeted strategies to prevent poultry colibacillosis and mitigate the public health risks of drug‐resistant strains entering the human food chain.

## 1. Introduction

Avian colibacillosis is one of the most commonly occurring bacterial diseases caused by avian pathogenic *Escherichia coli* (APEC), which results in serious losses to the poultry industry around the world [1]. Different APEC serotypes cause local or systemic infections in poultry, such as pericarditis and perihepatitis. Chickens around 1 month old had a higher incidence of disease, but they could be infected as early as 1 day old [2]. Although both raised chickens and adult chickens teach chicks to develop strong resistance, infections can still lead to symptoms such as salpingitis [3]. Currently, commercial *E. coli* vaccines are available; however, their effectiveness is limited due to the large number of *E. coli* serotypes and the lack of cross‐protection among them [4]. Furthermore, the existence of drug‐resistant bacteria and the ever‐increasing number of drug‐resistant strains over time are also becoming a great threat to chickens [5].

The extensive and indiscriminate use of antibiotics has become a significant driver in the development of bacterial drug resistance. The widespread use of antibiotics as growth promoters and prophylactic agents in the poultry breeding industry drives the accumulation and spread of diverse drug resistance genes [6]. In particular, broad‐host‐range conjugative plasmids that carry antibiotic resistance genes (ARGs) are critical for the spread of antibiotic resistance. Consequently, bacterial populations give rise to multidrug‐resistant (MDR), extensively drug‐resistant (XDR), and even pandrug‐resistant (PDR) strains [7].

Research data have indicated a significant increase in drug resistance among APEC over the past decade [8]. This trend has led to treatment failures in poultry, and poultry bacteria may transfer this acquired ability to human bacteria, which can disrupt the treatment of human infections [9]. For example, the *MCR-1* gene, which confers resistance to polymyxins, has been identified in APEC [10] and subsequently detected in pathogenic bacteria associated with humans [11]. This occurrence underscores the possible public health dangers linked to antibiotic‐resistant bacteria that come from animals [12].

In this study, we characterized one isolate, SDLYRA415. It was resistant to 28 antibiotics and displayed intermediate resistance to polymyxin B and antibiotic amikacin, which establishes its “extensively drug resistant” status. Furthermore, whole‐genome sequencing, conjugation experiment, and animal experiments were performed to investigate the characteristics of the XDR strain.

## 2. Methods

### 2.1. Bacterial Isolation and Identification

In April 2025, a large number of broilers belonging to a commercial indigenous chicken farm in the Shandong province of China displayed typical lesions of *E. coli* infections, and about 20% of chickens passed away. Brain tissue samples were aseptically collected, inoculated onto blood agar plates, and grown at 37°C. Single colonies were cultivated in BHI medium containing 5% chicken serum. Then the 27F/1492R primer pair was used to amplify the 16S rRNA sequence, and the PCR product was subsequently sequenced.

### 2.2. Growth Curve and Drug Susceptibility

For the determination of bacterial growth curves, bacterial strains were grown in liquid Luria‐Bertani (LB) media overnight at 37°C. The next day, overnight cultures were diluted 1:100 in fresh liquid LB media, which were then placed in 12‐well plates with three replicates per strain. Bacterial growth was monitored over 24 h at 37°C with shaking at 250 rpm using a microbial growth curve analyzer. Sampling occurred every 30 min with a 5 s delay at a wavelength of 600 nm (OD_600_). The data were then used to plot the isolate’s growth curve. The antimicrobial susceptibility analysis was carried out using the disk diffusion method. Briefly, the isolates were inoculated on LB agar, and the antimicrobial discs were dropped on the agar and incubated at 37°C for 12 h. The minimum inhibitory concentration (MIC) was performed using AutoMic‐i600 Automated Microbial Identification and Antimicrobial Susceptibility Testing System. *E. coli* ATCC 25922 was used for quality control of the experiment. The results of the drug sensitivity test were mainly measured in accordance with the Clinical and Laboratory Standards Institute (CLSI) M100‐ED35 standard guidelines (Supporting Information 1: Tables S1, S2). The CLSI breakpoints are from human breakpoints. The MIC result should be prioritized in cases where there is a disagreement between the disk diffusion method and the MIC method for antimicrobial susceptibility testing.

### 2.3. Genomic Analysis—the Sequencing, Assembly, and Annotation of the Genomes

The SDLYRA415 strain was cultured to log phase in liquid medium and shipped cold to Shanghai Personal Biotechnology Co., Ltd. (Personalbio) for whole‐genome sequencing. Genomic DNA was extracted via the CTAB method, with libraries prepared using Illumina’s TruSeq DNA Sample Preparation Kit and Pacific Biosciences’ Template Prep Kit. Sequencing was performed on Nanopore PromrthION48/Pacific Biosciences and Illumina Novaseq platforms. Personalbio conducted de novo assembly by first filtering data with AdapterRemoval (v2.2.2) and SOAPec (v2.03) to remove adapters and contaminants, then assembling third‐generation reads with HGAP (v4) to generate contigs, integrating results for a complete sequence, and refining it using Pilon (v1.18). They also performed comprehensive genome annotation, including predictions of protein‐coding genes, noncoding RNAs, CRISPRs, prophages, virulence genes, and CAZys, along with functional annotations against NR, eggNOG, KEGG, GO, and Swiss‐Prot databases.

We then used Snippy to identify single nucleotide polymorphisms (SNPs) between SDLYRA415 and the reference genome (Supporting Information 2: Supplementary data 1). For multistrain comparative genomic and phylogenetic analyses, snippy‐core was employed to extract “core SNPs” (located in fully covered, indel‐free regions across all samples) into a VCF file. This core SNP dataset was converted to a distance matrix using vcf2dis [13], which also generated a Newick‐format phylogenetic tree, visualizable with tools like MEGA. The Genome Taxonomy Database toolkit [14] was used to calculate average nucleotide identity (ANI) between SDLYRA415 and reference genome. Plasmid visualization and comparison with reference plasmids from NCBI were performed using the BLAST Ring Image Generator (BRIG) [15]. Plasmids were detected using PlasmidFinder (https://cge.food.dtu.dk/services/PlasmidFinder/). Strain multilocus sequence typing (MLST) typing was determined with MLST software (https://github.com/tseemann/mlst). Serotype of the strains by using https://cge.food.dtu.dk/services/SerotypeFinder. We used the progressive Mauve program in Mauve 2.3.1 software to compare the plasmid pSDLYRA415‐1 and reference plasmids to obtain a synteny relationship between the plasmids.

### 2.4. Conjugation Assay

For antibiotic‐resistant plasmids, rifampin‐resistant *E. coli* strain EC600 was used as the recipient. SDLYRA415 and EC600 were cultured to the logarithmic growth phase at 37°C in LB medium. Subsequently, 1 mL of SDLYRA415 and 1 mL of recipient EC600 cells were combined. The resulting mixture was washed with 4°C PBS, resuspended in LB broth, spread on 0.22 μm filter disks on LB agar plates, and incubated at 37°C for 12 h. Then the bacterial cultures were harvested and subjected to serial dilution. The diluted cultures were then plated on agar containing 5 IU/mL polymyxin B and 50 μg/mL rifampin to facilitate the detection of the *MCR-1*‐carrying SDLYRA415 Plasmid pSDLYRA415‐1.

To calculate the conjugation efficiency, the diluted culture was spread on plates containing only 50 μg/mL rifampin to determine the total number of recipient cells. The conjugation efficiency was calculated by dividing the number of transconjugants by the number of recipient cells. The conjugation experiments were performed in triplicates with three parallels in each biological replicate. To investigate the drug resistance of recipient cells, a total of three single bacterial colonies were randomly collected from the above dual‐antibiotic plates. To determine which plasmid had undergone transfer, we designed specific primers for each of the four plasmids (Table 1). The donor bacteria, recipient bacteria, and three positive conjugates were tested separately.

**Table 1:** Primers for each of the four plasmids.

Plasmid	Gene	Primers	Length (bp)
pSDLYRA415‐1	*MCR-1*	F: CTCGGTCAGTCCGTTTGT	679
		R: TGGCTTACGCATATCAGG	
pSDLYRA415‐2	*NovA*	F: GATTTCAGTCACTTCGTCG	922
		R: AGGCGGTAACAAAGGTAT	
pSDLYRA415‐3	*NDM-5*	F: GGACCGATGACCAGACCG	350
		R: CAGGCAGCCACCAAAAGC	
pSDLYRA415‐4	*Sul2*	F: CTTGCGGTTTCTTTTAGC	792
		R: GGCATCGTCAACATAACCTCGGACA	

### 2.5. Animal Experiment

All animal experiments were approved by Linyi University’s Institutional Animal Care and Use Committee (LYU20250117). Twenty 8‐week‐old female BALB/c mice were acclimated to their environment for 1 week prior to receiving a 1‐week course of four antibiotics (consisted of 1 g/L ampicillin sodium, 1 g/L neomycin sulfate, 1 g/L metronidazole, and 0.5 g/L vancomycin hydrochloride administered at a volume of 10 mL/kg body weight) administered via gavage, followed by antibiotic water for 3 days. The *E. coli* strain was cultured to the late log phase and resuspended in PBS to yield 1 × 10^9^ CFU/mL. In the infected group (*N* = 10), 200 μL *E. coli* strain was carried out by intraperitoneal injection into the mice. In the control group (*N* = 10), 10 mice were injected with PBS via the same route. Twenty 1‐day‐old commercial local breed chickens without a history of vaccination were purchased from a local farm and randomly divided into two groups. In the infected group, 10 birds at 5 days were inoculated with intraperitoneal injection with 200 μL of the isolated bacterial stock containing 1 × 10^9^ CFU/mL. In the control group, 10 birds were injected with the PBS via the same route. Mice and birds of all groups were maintained separately in isolators for 2 weeks with ad lib access to commercial feed and drinking water. Observe and record the incidence of diseases and death in animals during the feeding process. Animals were considered dead when breathing and heartbeat stopped permanently, pupils were fixed and dilated without a light reflex, and all reflexes were completely lost. The liver and heart were collected from the animal to determine bacterial loads and evaluate histopathologic changes, as described previously [16].

### 2.6. Statistical Analysis

Within the framework of animal pathogenicity experiments, survival analysis was conducted using Graphpad Prism8 Kaplan–Meier curves, supplemented by a log‐rank test to enable statistical comparison of mortality rates across various groups. To evaluate conjugation efficiency, 95% confidence intervals were calculated to accompany the point estimate. Results are expressed as means (± SE). Additionally, chi‐square test and Bonferroni‐corrected Kruskal–Wallis *H* test were utilized to compare among donor, recipient, and transconjugant strains in the examination of resistance profiles. *p* < 0.05 was considered statistically significant.

## 3. Results

### 3.1. Identification and Characterization of *E. coli* SDLYRA415

16S rRNA sequencing confirmed that the strains belonged to the *E. coli* (*data* not shown). The growth rates of SDLYRA415 were monitored under 37°C conditions. SDLYRA415 exhibited a lag phase from 0 to 1 h, a logarithmic phase from 1.5 to 15 h, and a stationary phase from 15.5 to 24 h (Supporting Information 3: Figure S1). Antimicrobial susceptibility test results for the *E. coli* isolate are shown in Tables 2 and 3. SDLYRA415 was resistant to 32 antibiotics, displayed only susceptibility to tigecycline, and had intermediate resistance to polymyxin B and amikacin. This result suggested that SDLYRA415 belonged to the XDR strain.

**Table 2:** Antimicrobial susceptibility profile of the donor, recipient, and transconjugant strains.

Susceptibility disk	Antibacterial diamet (mm) drug resistance				
	SDLYRA415	EC600	EC600‐1	EC600‐2	EC600‐3
Ampicillin (10 μg)	0 (R)	16 (I)	0 (R)	0 (R)	0 (R)
Piperacillin (100 μg)	0 (R)	20 (I)	0 (R)	11 (R)	0 (R)
Cephalothin (30 μg)	0 (R)	16 (I)	0 (R)	0 (R)	0 (R)
Cefazolin (30 μg)	0 (R)	22 (S)	0 (R)	0 (R)	0 (R)
Cefuroxime (30 μg)	0 (R)	20 (I)	0 (R)	0 (R)	0 (R)
Ceftriaxone (30 μg)	0 (R)	26 (S)	0 (R)	0 (R)	0 (R)
Cefotaxime (30 μg)	0 (R)	27 (S)	0 (R)	0 (R)	0 (R)
Ceftazidime (30 μg)	0 (R)	22 (S)	0 (R)	0 (R)	0 (R)
Cefoperazone (75 μg)	0 (R)	25 (S)	0 (R)	0 (R)	0 (R)
Cefepime (30 μg)	0 (R)	23 (S)	8 (R)	11 (R)	8 (R)
Gentamicin (10 μg)	0 (R)	22 (S)	14 (R)	22 (S)	13 (R)
Streptomycin (10 μg)	0 (R)	12 (I)	0 (R)	0 (R)	0 (R)
Kanamycin (30 μg)	0 (R)	20 (S)	0 (R)	20 (S)	0 (R)
Tobramycin (10 μg)	0 (R)	15 (I)	0 (R)	15 (I)	0 (R)
Tetracycline (30 μg)	0 (R)	20 (S)	12 (I)	20 (S)	0 (R)
Chloramphenicol (30 μg)	0 (R)	20 (S)	0 (R)	14 (I)	0 (R)
Norfloxacin (10 μg)	0 (R)	18 (S)	9 (R)	9 (R)	0 (R)
Ofloxacin (5 μg)	0 (R)	18 (S)	13 (I)	16 (S)	0 (R)
Polymyxin B (300 IU)	10 (I)	17 (S)	13 (S)	13 (S)	11 (I)
Aztreonam (30 μg)	0 (R)	24 (S)	10 (R)	21 (I)	0 (R)
Cefoxitin (30 μg)	0 (R)	23 (S)	0 (R)	0 (R)	0 (R)
Spectinomycin (100 μg)	0 (R)	15 (I)	0 (R)	0 (R)	0 (R)
Imipenem (10 μg)	0 (R)	22 (I)	0 (R)	0 (R)	0 (R)
Meropenem (10 μg)	0 (R)	22 (I)	0 (R)	0 (R)	0 (R)
Amikacin (30 μg)	15 (R)	18 (I)	18 (I)	18 (I)	13 (R)
Ciprofloxacin (5 μg)	0 (R)	18 (R)	13 (R)	13 (R)	0 (R)
Levofloxacin (5 μg)	0 (R)	17 (I)	13 (R)	13 (R)	0 (R)
Compound‐sulfamethoxazole (1.25 μg/23.75 μg)	0 (R)	21 (S)	0 (R)	0 (R)	0 (R)
Amoxicillin (25 μg)	0 (R)	18 (S)	0 (R)	0 (R)	0 (R)
Cefoperazone‐sulbactam (75 μg/75 μg)	0 (R)	23 (S)	0 (R)	0 (R)	0 (R)

**Table 3:** MIC value between the donor, recipient, and transconjugant strains.

Susceptibility disk	MIC value (μg/mL) drug resistance				
	SDLYRA415	EC600	EC600‐1	EC600‐2	EC600‐3
Ampicillin	>32 (R)	≤8 (S)	>32 (R)	>32 (R)	>32 (R)
Cefazolin	>32 (R)	≤2 (S)	>32 (R)	>32 (R)	>32 (R)
Cefuroxime	>16 (R)	8 (I)	>16 (R)	>16 (R)	>16 (R)
Cefotaxime	>4 (R)	≤0.12 (S)	>4 (R)	>4 (R)	>4 (R)
Ceftazidime	>16 (R)	≤1 (S)	>16 (R)	>16 (R)	>16 (R)
Cefepime	>16 (R)	≤0.5 (S)	>16 (R)	>16 (R)	>16 (R)
Gentamicin	8 (R)	≤1 (S)	4 (I)	≤1 (S)	8 (R)
Tetracycline	>16 (R)	≤2 (S)	>16 (R)	≤2 (S)	>16 (R)
Aztreonam	>16 (R)	≤4 (S)	>16 (R)	≤4 (S)	>16 (R)
Cefoxitin	>32 (R)	≤8 (S)	>32 (R)	>32 (R)	>32 (R)
Imipenem	4 (R)	≤0.25 (S)	16 (R)	8 (R)	4 (R)
Meropenem	8 (R)	≤0.06 (S)	16 (R)	16 (R)	4 (R)
Amikacin	8 (I)	≤2 (S)	8 (I)	≤4 (S)	8 (I)
Levofloxacin	>8 (R)	0.25 (S)	1 (I)	1 (I)	>8 (R)
Tigecycline	≤0.25 (S)	≤0.25 (S)	≤0.25 (S)	≤0.25 (S)	≤0.25 (S)
Compound‐sulfamethoxazole	>4/76 (R)	≤0.5/9.5 (S)	>4/76 (R)	>4/76 (R)	>4/76 (R)
Cefoperazone‐sulbactam	>64/32 (R)	≤16/8 (S)	>64/32 (R)	>64/32 (R)	>64/32 (R)
Ampicillin‐sulbactam	>32/16 (R)	≤8/4 (S)	>32/16 (R)	>32/16 (R)	>32/16 (R)
Ceftazidime‐avibactam	>16/4 (R)	≤0.5/4 (S)	>16/4 (R)	>16/4 (R)	>16/4 (R)
Piperacillin‐tazobactam	>64/4 (R)	≤8/4 (S)	>64/4 (R)	>64/4 (R)	>64/4 (R)
Amoxicillin‐clavulanate	>32/16 (R)	≤8/4 (S)	>32/16 (R)	>32/16 (R)	>32/16 (R)

### 3.2. Whole‐Genome Analysis

**Figure 1:**
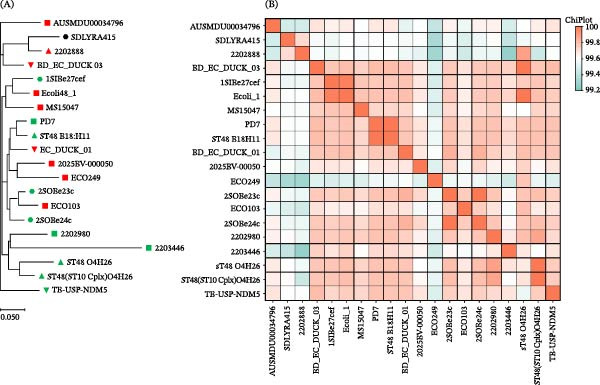
Single nucleotide polymorphism evolutionary and average nucleotide identity analysis of SDLYRA415 and ST48 *E. coli* reference strain. (A) The SNP evolution tree was observed using MEGA11. •Isolates in this study, 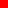
human isolates, 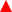
broiler chicken isolates, 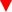
duck isolates, 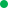
wastewater treatment plant (WWTP) isolates, 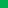
pig isolates, 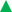
fruit bat isolates, and 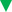
animal gut isolates. (B) Average nucleotide identity results.

The 5.51 Mb complete genome of *E. coli* SDLYRA415 has a total guanine and cytosine (GC) content of 50.61% with a single chromosome and four plasmids (Table 4, Supporting Information 3: Figure S2A–E). Given the genome sequences, we performed in phylotyping, MLST genotyping, serotyping, and PlasmidFinder for *E. coli* SDLYRA415 genome as described. The phylogroup of *E. coli* SDLYRA415 is A, the MLST genotype is ST48, and the serotyping is O6: H16. The ANI values between SDLYRA415 and all the *E. coli* ST48 reference strains were 99.27%−99.71% (Figure 1B). The highest ANI value between SDLYRA415 and the reference genome was between SDLYRA415 and 220288, with 99.71% ANI. The SNP phylogeny was consistent with the results of MLST typing (Figure 1A), indicating that SDLYRA415 had the closest relative to 220288 (isolated from broilers in the Netherlands).

**Table 4:** Genome summary for SDLYRA415.

Replicon	Sequence length (bp)	Plasmid rep type (s)	Antimicrobial resistance gene
SDLYRA415 chromosome	4,920,759	NA	*bla*_CTX-M−55_
pSDLYRA415‐1	247,169	IncHI2, IncHI2A, IncN	*MCR-1*, *bla*_CTX-M−65_
pSDLYRA415‐2	159,662	IncFIB (AP001918), IncFIC(FII)	—
pSDLYRA415‐3	113,650	—	*NDM-5*
pSDLYRA415‐4	77,412	p0111	—

**Figure 2:**
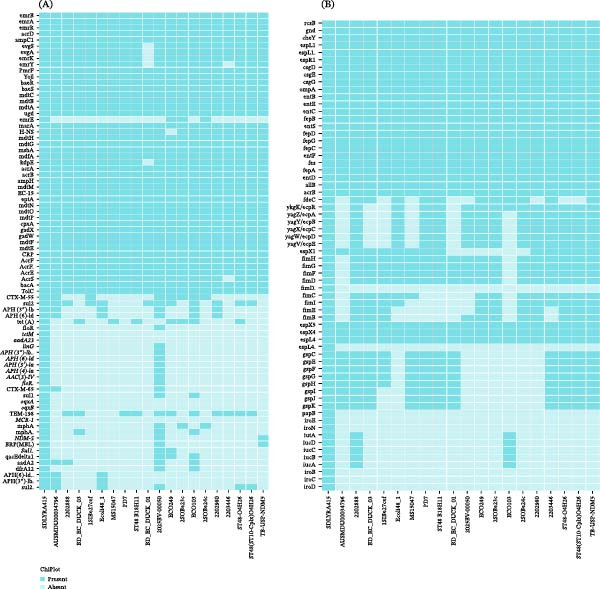
Binary distribution profiles of antibiotic resistance genes and virulence genes among ST48 *E. coli* strains. (A) Resistance gene binary map: the left column lists antibiotic resistance gene subtypes, and the bottom row lists ST48 *E. coli* strains (including SDLYRA415 and reference strains). (B) Virulence gene binary map: the left column lists virulence factor gene subtypes, and the bottom row lists ST48 *E. coli* strains. “Present” (colored) indicates the presence of the corresponding virulence gene, while “Absent” (blank/white) indicates the absence of the gene.

Comprehensive Antibiotic Resistance Database (CARD) analysis identified 78 ARGs (*E*‐value <1e^−10^, identity ≥90%, query cover ≥90%) (Supporting Information 2: Supplementary data 2) in *E. coli* SDLYRA415, 52 ARGs located to the chromosome (including *bla*_CTX-M-55_), 16 to the plasmid 1 (including *MCR-1*, *bla*_CTX-M-65_, and *bla*_TEM-198_),7 to the plasmid 3 (including *NDM-5*) and 3 to the plasmid 4. Among the reference strains, 2025BV‐00050 showed the highest similarity to SDLYRA415, with a similarity percentage of 89.3%, whereas BD_EC_DUCK_01 showed the lowest similarity, 52.0%. All strains collectively possessed 36 conserved resistance genes, primarily associated with fundamental bacterial resistance mechanisms, such as efflux pump genes including emrB, acrA, and marA (Figure 2A, Supporting Information 2: Supplementary data 2). Notably, SDLYRA415 uniquely contained the *MCR-1* gene and concurrently harbored low‐prevalence genes such as *bla*_CTX-M-55_, *bla*_CTX-M-65_, and *NDM-5*, which confer resistance to colistin, β‐lactams, and carbapenems, respectively. These genes are the key molecular components that underpin its extensive drug‐resistant phenotype.

A total of 64 virulence factors were identified using the Virulence Factor Database (VFDB) with strict criteria (*E*‐value ≤1e^−50^, sequence identity ≥90%, query coverage ≥90%) in *E. coli* SDLYRA415, 54 virulence factor genes are located on the chromosome and 10 to the plasmid 2 (Supporting Information 2: Supplementary data 3). Among the reference strains, strain 2202888 showed the highest virulence gene similarity (87.5%) to SDLYRA415, coharboring 56 virulence genes, while ECO103 exhibited the lowest similarity (53.1%) with only 34 virulence genes detected (Figure 2B, Supporting Information 2: Supplementary data 3). A total of 27 conserved virulence genes were shared by all strains, primarily involved in basic bacterial pathogenic functions (e.g., rcsB and ompA). Notably, SDLYRA415 uniquely harbored eight virulence genes, including fimD, espL4, papB, iroE, iroN, iroB, iroC, and iroD. Among these, the iro gene cluster (iroB, iroC, iroD, iroE, iroN) is involved in siderophore synthesis and iron acquisition, which may enhance the strain’s survival competitiveness within the host, while papB and fimD are associated with bacterial adhesion capability—a key initial step in pathogenic colonization.

**Figure 3:**
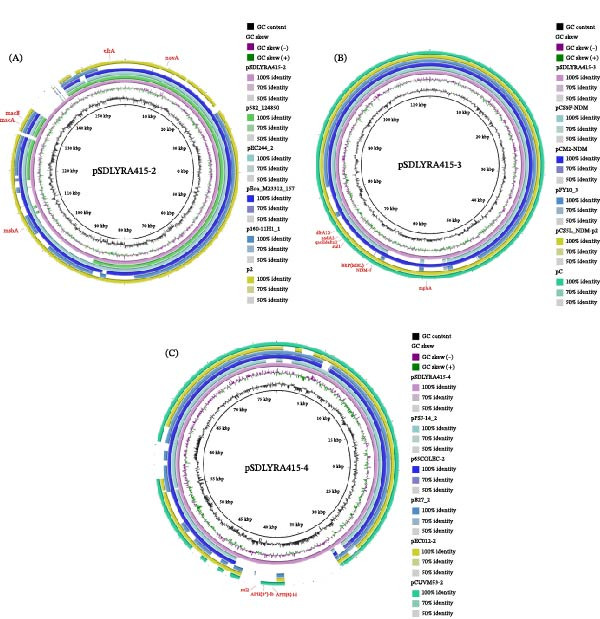
Comparison of the plasmid and the reference plasmid using BRIG. (A) pSDLYRA415‐2 and p582_124850 (GenBank: CP120569.1), pEC244_2 (GenBank: CP019018.1), pEco_M23312_157 (GenBank: CP133945.1), p160‐11H1_1 (GenBank: AP031376.1), and p2 (GenBank: CP164284.1). (B) pSDLYRA415‐3 and pCS9F‐NDM (GenBank: CP158451.1), pCM2‐NDM (GenBank: CP158309.1), pFY10_3 (GenBank: CP095819.1), pCS5L_NDM‐p2 (GenBank: CP158312.1), and pC (GenBank: CP069173.1). (C) pSDLYRA415‐4 and pPSJ‐14_2 (GenBank: AP027628.1), p65COLEC_2 (GenBank: CP070916.1), pB27_2 (GenBank: AP027426.1), pEC012_2 (GenBank: CP119579.2), and pCUVM53_2 (GenBank: CP116978.1).

PlasmidFinder identified plasmid 1 belonging to IncHI2, IncHI2A, and IncN group. Plasmid 2 belonging to IncFIB(AP001918) and IncFIC(FII) group. Plasmid 4 belonged to p0111 group, and no known plasmid type was matched to plasmid 3 (Table 4), indicating that plasmid 3 belonged to a novel plasmid type. BLAST analysis of the *E. coli* SDLYRA415 plasmids identified in this study showed that three of the four plasmids were similar to previously reported plasmids in the NCBI sequence database. Plasmids pSDLYRA415‐2, pSDLYRA415‐3, pSDLYRA415‐4 showed 61%–94%, 74%–100%, 74%–82% sequence coverage separately (Figure 3A–C), indicating that these similar plasmids were detected previously by other researchers. We did not identify plasmid sequences with high similarity to plasmid pSDLYRA415‐1 in the NCBI database.

### 3.3. Characterization of *MCR-1* and *bla*_CTX-M−55_ Bearing Plasmid pSDLYRA415‐1

The *bla*_CTX-M-55_ and *MCR-1* cobearing plasmid pSDLYRA415‐1 was 247, 169 bp in size and had an average GC content of 47%. The results of annotation indicated that pSDLYRA415‐1 harbored 279 open reading frames (ORFs) and was a hybrid plasmid containing multiple replicons, including IncHI2, IncHI2A, and IncN. CARD analysis identified a number of ARGs in pSDLYRA415‐1, including the tetracycline resistance gene *tetM*; aminoglycoside resistance genes *aadA23*, *APH*(*3*″)*-Ib*, *APH*(*6*)*-Id*, *APH*(*3*′)*-Ia*, *APH*(*4*)*-Ia*, and *AAC*(*3*)*-IV*; lincosamide resistance gene *linG*; chloramphenicol resistance gene *floR*; β‐lactam resistance genes *bla*_CTX-M-55_, *bla*_TEM-198_; sulfonamide resistance genes *sul1*; fluoroquinolone resistance genes *oqxA* and *oqxB*; polymyxin resistance gene *MCR-1*; and macrolide resistance genes mphA.

**Figure 4:**
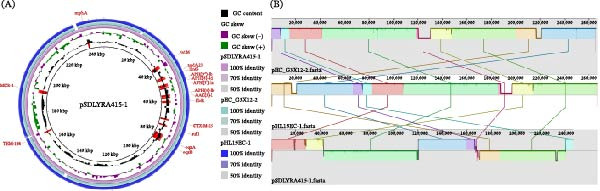
Comparison of the plasmid pSDLYRA415‐1 and the reference plasmid using BRIG and Mauve. (A) pSDLYRA415‐1 and pEC_G3X12‐2 (GenBank: NZ_ON960342) and pHL15EC‐1 (GenBank: CP079769.1). (B) The modules of identical color, linked by lines, signify the collinear segments between the genomes, indicating the absence of genomic rearrangement within these regions. Conversely, regions outside the similarly colored areas suggest a lack of detectable homology between the input genomes. The entirely white regions within a fragment denote the absence of alignment between the genomes, potentially indicating the presence of unique components or mutations.

BLASTN alignment showed that the first 50% of the pSDLYRA415‐1 sequence length has 100% query cover and 99.97% similarity to the pHL15EC‐1 plasmid, the last 50% of the pSDLYRA415‐1 sequence length has 97% query cover and 100.00% similarity to the pEC_G3X12‐2 plasmid. The synteny between pSDLYRA415‐1, pHL15EC‐1 and pEC_G3X12‐2 was conducted using Mauve software. The results are shown in Figure 4A,B. There were many local collinear blocks (LCBs) between the plasmids. In addition, there were insertions and deletions between the plasmids and many genome rearrangement events, such as translocations and inversions.

### 3.4. Transfer of Antibiotic Resistance

SDLYRA415 successfully transferred polymyxin B resistance to EC600 by conjugation at rates of 6.5 × 10^−7^ (± 0.264 × 10^−7^), with a 95% confidence interval 5.85 × 10^−7^−7.15 × 10^−7^. Antimicrobial susceptibility assay results showed that the three recipient cells (EC600‐1,2,3) had different antibacterial susceptibility profiles. EC600 was sensitive to 28 antibiotics and displayed intermediate resistance to 6 antibiotics. EC600‐1 was resistant to 30 antibiotics and sensitive to polymyxin B and tigecycline. EC600‐2 was resistant to 25 antibiotics, and EC600‐3 was resistant to 32 antibiotics and displayed sensitivity to tigecycline. The results of statistical analysis showed significant differences in resistance rates between the recipient and all transconjugant strains (*p* < 0.001), indicating effective horizontal transfer of resistance plasmids (Tables 2 and 3). EC600‐3 had a similar resistance rate to the donor SDLYRA415 (*p* > 0.05), suggesting full acquisition of resistance. However, EC600‐1 and EC600‐2 had lower resistance rates than EC600‐3 (*p* < 0.05 and *p* < 0.01), indicating varying levels of plasmid transfer completeness among transconjugants (Tables 2 and 3). PCR confirmed that EC600‐1 contained plasmid 1 and plasmid 3, EC600‐2 contained plasmid 1 and plasmid 3, and EC600‐3 contained plasmid 1, plasmid 2, plasmid 3, and plasmid 4 (Supporting Information 3: Figure S3).

### 3.5. Pathogenicity of *E. coli* SDLYRA415 in Mice and Chickens

**Figure 5:**
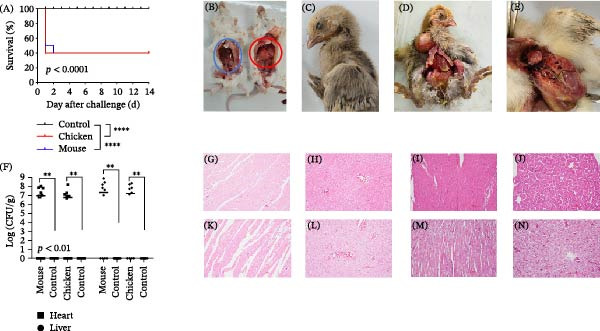
Results of animal experiments. (A) 10 birds and mice were inoculated intraperitoneal injection with 200 μL of the isolated bacterial stock containing 1 × 10^9^ CFU/mL. Survival was monitored daily. (B) Hepatomegaly in mice, blue circles indicate livers from the control group, red circles indicate livers from the experimental group. (C) Nasal discharge. (D) Perihepatitis. (E) Pericarditis. (F) Bacterial loads in the heart and liver of infected chickens and mice (log_10_ CFU/g tissue). (G) Normal heart tissue of control mice, (H) normal liver tissue of control mice, (I) normal heart tissue of control chickens, (J) normal liver tissue of control chickens, (K) disorganized myocardial fibers with interstitial inflammatory cell infiltration in infected mice, (L) obscured hepatic lobules, hepatocellular degeneration/necrosis, and hepatic sinus obstruction in infected mice, (M) disorganized myocardial fibers with interstitial edema and congestion in infected chickens, and (N) obscured hepatic lobules, hepatocellular degeneration/necrosis accompanied by inflammatory cell infiltration in infected chickens. Hematoxylin and eosin (H & E) staining; scale bars = 100 μm. ^∗∗^ *p* < 0.01,  ^∗∗∗∗^ *p* < 0.0001.

We first investigated the pathogenicity of *E. coli* SDLYRA415 in mice by intraperitoneal injection. Some mice in the challenge group exhibited clinical signs, including lethargy and ruffled, erect fur (piloerection), prior to death. Within a 24 h period, five mice died, followed by another death at ~30 h, resulting in a total mortality of six (Figure 5A). *E. coli* SDLYRA415 significantly increased mice mortality compared with the control (*p* < 0.0001). Gross examination revealed marked hepatomegaly in deceased animals (Figure 5B). We then investigated the pathogenicity of *E. coli* SDLYRA415 in chickens. Of 10 chicks, 6 demonstrated depression and died suddenly at less than 24 h postinoculation (Figure 5A). *E. coli* SDLYRA415 significantly increased chickens’ mortality compared with the control (*p* < 0.0001). Over the subsequent 2 days, three out of the remaining four subjects exhibited symptoms of mental exhaustion, reluctance to stand, and limping. By the fourth day postchallenge, all four chickens had reverted to their normal condition and survived to the end of the experiment. Birds that succumbed to the challenge had lesions such as pericarditis, perihepatitis, and nasal discharge (Figure 5C–E). All mock‐infected control mice and chickens did not develop any clinical signs and survived until the end of the observation period.

The colony count results demonstrate that the hearts and livers of animals, both mice and chickens, that succumbed to infection exhibited elevated levels of *E. coli* (Figure 5F). Conversely, no *E. coli* was detected in the hearts and livers of surviving animals or those in the control group. Subsequently, we performed further histopathological observations on the heart and liver. Microscopic examination of heart and liver sections from the control group revealed a normal histological structure (Figure 5G–J). Mice infected with *E. coli* exhibited disorganization and degeneration of myocardial fibers with infiltration of interstitial inflammation (Figure 5K), and changes in hepatic lobules, degeneration, or necrosis of hepatocytes (Figure 5L). Chickens infected with *E. coli* exhibited pronounced cardiac and hepatic lesions, characterized by disorganized myocardial fibers with interstitial edema and congestion (Figure 5M), as well as obscured hepatic lobules, hepatocellular degeneration, and necrosis accompanied by inflammation (Figure 5N).These results suggest that acute multiorgan inflammation was the underlying cause of mouse and chicken mortality.

## 4. Discussion

APEC, a subgroup of extraintestinal pathogenic *E. coli* (ExPEC), causes avian colibacillosis and imposes economic losses on the poultry industry worldwide [17]. The high proportion of multidrug‐resistance phenomena is prevalent in APEC and environmental strains [18]. XDR‐APEC strains have been reported periodically. These XDR strains frequently contain gene clusters like *MCR*, *bla*, *oqx*, and *floR*, which can be transferred horizontally, aiding their dissemination among birds, humans, and the environment [19]. This propagation poses significant challenges for veterinary and clinical medicine by drastically reducing available therapeutic options.

Notably, SDLYRA415 shows distinct genotypic and phenotypic features compared with most common APEC strains. Globally, dominant APEC strains mainly belong to serogroups O78, O2, and O117 and high‐risk sequence types ST117, ST140, and ST95 [20], while SDLYRA415 belongs to rare serotype O6:H16, phylogroup A, and ST48, a type traditionally considered as commensal or low‐virulence avian *E. coli* [21]. This rare ST48‐O6:H16 background coupled with XDR and high pathogenicity makes it an emerging high‐risk clone rarely reported in poultry.

Significantly, the resistance genes are organized on a single chromosome and four plasmids. Of particular interest is the IncHI2/IncN hybrid plasmid, referred to as pSDLYRA415‐1, which concurrently carries the *MCR-1* and *bla*_CTX-M-55_ genes. IncHI2 plasmids are distinguished by their broad host range and high conjugation efficiency [22]. While these plasmids have been previously identified in Salmonella [23], their presence in this APEC strain highlights livestock bacteria as a key vector for the one‐step horizontal transfer of carbapenemases, polymyxin resistance, and ESBLs to human‐related bacteria. Additionally, plasmid pSDLYRA415‐3 is a unique unclassified replicon that shows no homology to known plasmid types and harbors the clinically essential carbapenem resistance gene *NDM-5* [24]. This plasmid likely emerged via the modular recombination of mobile genetic elements in microbial communities found on poultry farms, further increasing the risk of carbapenem resistance spreading between species and posing greater public health risks [25].

There are notable differences in the abundance of resistance genes and core determinants when comparing SDLYRA415’s resistome with recently reported XDR‐APEC strains. For example, the XDR‐APEC strain JS01 (ST155/O177:H51) contains 58 resistance genes, including *bla*_CTX-M-14_ and *MCR-3*, but does not have carbapenemase genes such as *NDM-5* [26]. Another XDR‐APEC strain carries *MCR-1*, *bla*_TEM-176_, and *bla*_CTX-M-14_ within 42 resistance genes but is without *bla*_CTX-M-55_ and *NDM-5* [11]. SDLYRA415, in contrast, has 78 resistance genes, a number much higher than those in recent studies [18], and includes a distinct combination of *bla*_CTX-M-55_, *bla*_CTX-M-65_, *MCR-1*, and *NDM-5*, not seen in other XDR‐APEC strains. With its unique plasmid repertoire, this distinct resistome increases SDLYRA415’s adaptability to antibiotic pressure and elevates public health risks through the cotransmission of important resistances.

Conjugation assays demonstrated that pSDLYRA415‐1 transferred polymyxin resistance to EC600 at a frequency of 6.5 × 10^−7^. While this absolute rate appears modest [27], the high stocking density, stress, and constant selective pressure from sub‐therapeutic antibiotics in poultry houses can raise the real‐world transfer probability [28]. Significantly, the three randomly selected transconjugants (EC600‐1, ‐2, and ‐3) acquired distinct plasmid combinations, leading to varied resistance profiles. This observation not only confirms the efficient transfer of the hybrid plasmid pSDLYRA415‐1, which harbors *MCR-1* and *bla*_CTX-M-55_, but also indicates that other plasmids, such as pSDLYRA415‐3 (containing *NDM-5*), are either self‐transmissible or capable of being mobilized.

Of particular concern is the acquisition of all four plasmids by transconjugant EC600‐3, which exhibited a resistance profile nearly identical to that of the original SDLYRA415 strain. This finding illustrates the potential real‐world risk of a “one‐step” emergence of pandrug or XDR bacteria in natural environments, such as the avian gut [29].This reflects the past use of various antibiotics as growth promoters [30], turning poultry plasmids into mobile multidrug reservoirs. This significantly hastens the emergence and spread of PDR clones, thereby undermining last‐resort therapeutic options. The appearance of a carbapenemase in APEC is alarming because ceftiofur, fluoroquinolones, and polymyxins still constitute the last‐resort arsenal in veterinary medicine [31]. Co‐occurrence of *NDM-5* and *MCR-1* abolishes β‐lactam options and drives elevated polymyxin usage, thereby reinforcing maintenance of *MCR-1* [32].

Animal experiments showed that the lethal rate of the strain in mice and day old chicks was 60%. Previous studies documented that certain APEC isolates induce mouse mortality. A yolk peritonitis‐derived chicken APEC strain caused 50% mouse mortality (2.985 × 10^7^ CFU/mL) with severe intestinal lesions [33]. ST155/O177:H51 APEC strain JS01 led to 100% mortality in mice within 72 h [26]. However, most mouse‐lethal APECs belong to high‐risk serotypes (e.g., O177:H51), whereas SDLYRA415—traditionally low‐virulent ST48‐O6:H16—induces 60% mouse mortality. This highlights its unusual cross‐species pathogenicity, likely linked to its virulence gene repertoire.

SDLYRA415 carries FimH, yagZ/ecpA and fdeC, which mediate stable bacterial adhesion and colonization in host tissues [34]. Furthermore, the aerobactin operon (iucABCD and iutA) and salmochelin cluster (iroBCDEN) enable efficient iron acquisition, supporting robust bacterial proliferation in vivo [35]. The ompA and rcsB genes enhance serum resistance and immune escape, avoiding host clearance [36]; espL4 and gsp cluster promote deep tissue invasion and inflammatory damage [24]. Together, these essential virulence factors cause quick systemic infections and acute visceral lesions, which consistently result in high lethality in animal models.

Elevated mortality rates in avian and mammalian models underline a substantial zoonotic threat, indicating that XDR‐APEC strains SDLYRA415 can move between species and pose a major public health risk. To mitigate the threats of XDR‐APEC to the poultry industry and public health, systematic and targeted control measures are urgently needed for commercial poultry farming. First, regular and targeted surveillance should be established in broiler farms to achieve early detection and early warning of high‐risk strains [37]. Second, the irrational use of antibiotics in poultry breeding must be strictly banned, and alternative products such as probiotics, prebiotics, and plant extracts can be used to replace antibiotics for disease prevention [38]. Third, strengthening farm biosecurity management is a key measure to block the horizontal transmission of drug‐resistant strains and plasmids [39].

While this study provides valuable insights into the resistance profiles and pathogenic mechanisms of the XDR‐APEC, it has several limitations. Only a single XDR‐APEC strain was isolated from one local broiler farm, so the regional prevalence and transmission potential of this high‐risk ST48 clone cannot be fully represented. Besides, the long‐term stability of its MDR plasmids in natural farm environments was not further investigated.

## 5. Conclusion

This study demonstrates that the APEC strain SDLYRA415 (ST48, O6:H16) is a significant XDR pathogen, harboring 78 ARGs on its chromosome and four plasmids, including *MCR-1*, *bla*_CTX-M-55_, *bla*_CTX-M-65_, and *NDM-5*. These genes, which promote the rapid spread of drug resistance, exist in the highly transferable hybrid plasmid pSDLYRA415‐1 and the novel plasmid pSDLYRA415‐3. SDLYRA415 caused 60% mortality in both chicken and mouse models, indicating its high pathogenicity. The coexistence of resistance genes, transferable plasmids, and avian virulence poses a serious threat to the poultry industry and public health, which requires attention.

## Author Contributions

Peikun Wang and Xinglin Zhang conceived the idea, completed the draft, provided the funding of research, and reviewed and approved the final manuscript. Weishi Ni performed the experiments and wrote the manuscript. Lijuan Chen, Hao Chen, and Kejing Wang revised the manuscript.

## Funding

This study was supported by the National Project for the Improvement of Animal and Plant Protection Capacity (Grant 20241000020103504) and the National Natural Science Foundation of China (Grant 32202810).

## Consent

The authors have nothing to report.

## Conflicts of Interest

The authors declare no conflicts of interest.

## Supporting Information

Additional supporting information can be found online in the Supporting Information section.

## Supporting information


**Supporting Information 1** Table S1: Breakpoints values/ranges for resistant (R), susceptible (S), and intermediate (I). Table S2: Breakpoints MIC values/ranges for resistant (R), susceptible (S), and intermediate (I).

**Supporting Information 2** Supplementary data1, Supplementary data2, Supplementary data3.

**Supporting Information 3** Figure S1: Growth curve of isolated strains. Growth curve of the APEC strain SDLYRA415 was determined by measuring the optical density at 600 nm (OD_600_) every 30 min for 24 h at 37°C in LB medium. The growth curve showed that SDLYRA415 exhibited a lag phase from 0 to 1 h, a logarithmic phase from 1.5 to 15 h, and a stationary phase from 15.5 to 24 h, indicating normal growth activity of the strain under laboratory conditions. Figure S2: Genome map of the SDLYRA415 chromosome and plasmids. (A) Circular genome map of the SDLYRA415 chromosome, showing the distribution and orientation of predicted coding sequences, COG functional annotation, GC content, and GC skew. The complete genome of SDLYRA415 is 5.51 Mb with a GC content of 50.61%, consisting of one chromosome and four plasmids. (B) Circular genome map of plasmid pSDLYRA415‐1, which belongs to IncHI2/IncHI2A/IncN replicon type. The map presents predicted coding sequences, GC content, GC skew and the location of key resistance genes including *MCR-1* and *bla*_CTX-M-55_. (C) Circular genome map of plasmid pSDLYRA415‐2, which belongs to IncFIB(AP001918)/IncFIC(FII) replicon type. The map displays predicted coding sequences, GC content, GC skew, and the distribution of virulence‐related genes. (D) Circular genome map of plasmid pSDLYRA415‐3, which is identified as a novel plasmid type with no known matched replicon. The map shows predicted coding sequences, GC content, GC skew, and the location of the carbapenem resistance gene *blaNDM-5*. (E) Circular genome map of plasmid pSDLYRA415‐4, which belongs to p0111 replicon type. The map illustrates predicted coding sequences, GC content and GC skew. Figure S3: PCR identification results of conjugated plasmid. Agarose gel electrophoresis was used to verify the transfer of four plasmids from donor strain SDLYRA415 to recipient strain EC600. M: DL5000 DNA marker (5000, 3000, 2000, 1500, 1000, 750, 500, 250, and 100 bp). 1: PCR product of pSDLYRA415‐1 from SDLYRA415 (positive control); 2: negative control of pSDLYRA415‐1; 3‐6: detection of pSDLYRA415‐1 in EC600, EC600‐1, EC600‐2, and EC600‐3; 7: PCR product of pSDLYRA415‐2 from SDLYRA415 (positive control); 8: negative control of pSDLYRA415‐2; 9‐12: detection of pSDLYRA415‐2 in EC600, EC600‐1, EC600‐2, and EC600‐3; 13: PCR product of pSDLYRA415‐3 from SDLYRA415 (positive control); 14: negative control of pSDLYRA415‐3; 15‐18: detection of pSDLYRA415‐3 in EC600, EC600‐1, EC600‐2, and EC600‐3; 19: PCR product of pSDLYRA415‐4 from SDLYRA415 (positive control); 20: negative control of pSDLYRA415‐4; 21‐24: detection of pSDLYRA415‐4 in EC600, EC600‐1, EC600‐2, and EC600‐3. The PCR results confirmed that pSDLYRA415‐1, pSDLYRA415‐2, pSDLYRA415‐3, and pSDLYRA415‐4 were successfully transferred into EC600 alone or in combination, which was consistent with the changes in drug resistance levels of transconjugants.

## Data Availability

This Whole Genome Shotgun project has been deposited at DDBJ/ENA/GenBank under the Accession Number JBRIOY000000000.
