# Supplementary material for: Genomic and Pathogenic Characterization of an Extensively Drug‐Resistant Avian Pathogenic Escherichia coli Strain
Source: Transbound Emerg Dis. 2026 May 14;2026:4233989. doi: 10.1155/tbed/4233989 (PMC13173369; doi:10.1155/tbed/4233989)
Supplement: Supplementary file 1 — Supporting Information 1 Table S1: Breakpoints values/ranges for resistant (R), susceptible (S), and intermediate (I). Table S2: Breakpoints MIC values/ranges for resistant (R), susceptible (S), and intermediate (I). [file TBED-2026-4233989-s002.doc]

**Table S1 Breakpoints values/ranges for Resistant(R), susceptible(S), and intermediate(I)**

|  | Susceptibility disk | R (mm) | I (mm) | S (mm) |
| --- | --- | --- | --- | --- |
| 1 | Ampicillin (10μg) | ≤ 13 | 14-16 | ≥ 17 |
| 2 | Piperacillin (100μg) | ≤ 17 | 18-20 | ≥ 21 |
| 3 | Cephalothin (30μg) | ≤ 14 | 15-17 | ≥ 18 |
| 4 | Cefazolin (30μg) | ≤ 14 | - | ≥ 15 |
| 5 | Cefuroxime (30μg) | ≤ 14 | 15-22 | ≥ 23 |
| 6 | Ceftriaxone (30μg) | ≤ 19 | 20-22 | ≥ 23 |
| 7 | Cefotaxime (30μg) | ≤ 22 | 23-25 | ≥ 26 |
| 8 | Ceftazidime (30μg) | ≤ 17 | 18-20 | ≥ 21 |
| 9 | Cefoperazone (75μg) | ≤ 15 | 16-20 | ≥ 21 |
| 10 | Cefepime (30μg) | ≤ 18 | 19-22 | ≥ 23 |
| 11 | Gentamicin (10μg) | ≤ 14 | 15-17 | ≥ 18 |
| 12 | Streptomycin (10μg) | ≤ 11 | 12-14 | ≥ 15 |
| 13 | Kanamycin (30μg) | ≤ 13 | 14-17 | ≥ 18 |
| 14 | Tobramycin (10μg) | ≤ 12 | 13-16 | ≥ 17 |
| 15 | Tetracycline (30μg) | ≤ 11 | 12-14 | ≥ 15 |
| 16 | Chloramphenicol (30μg) | ≤ 12 | 13-17 | ≥ 18 |
| 17 | Norfloxacin (10μg) | ≤ 12 | 13-16 | ≥ 17 |
| 18 | Ofloxacin (5μg) | ≤ 12 | 13-15 | ≥ 16 |
| 19 | Polymyxin B (300IU) | ≤ 8 | 9-11 | ≥ 12 |
| 20 | Aztreonam (30μg) | ≤ 17 | 18-20 | ≥ 21 |
| 21 | Cefoxitin (30μg) | ≤ 14 | 15-17 | ≥ 18 |
| 22 | Spectinomycin (100μg) | ≤ 14 | 15-17 | ≥ 19 |
| 23 | Imipenem (10μg) | ≤ 19 | 20-22 | ≥ 23 |
| 24 | Meropenem (10μg) | ≤ 19 | 20-22 | ≥ 23 |
| 25 | Amikacin (30μg) | ≤ 16 | 17-19 | ≥ 20 |
| 26 | Ciprofloxacin (5μg) | ≤ 21 | 22-25 | ≥ 26 |
| 27 | Levofloxacin (5μg) | ≤ 16 | 17-20 | ≥ 21 |
| 28 | Compound Sulfamethoxazole (1.25μg/23.75μg) | ≤ 10 | 11-15 | ≥ 16 |
| 29 | Amoxicillin (25μg) | ≤ 13 | 14-17 | ≥ 18 |
| 30 | Cefoperazone/Sulbactam (75μg/75μg) | ≤ 15 | 16-20 | ≥ 21 |

**Table S2 Breakpoints MIC values/ranges for Resistant(R), susceptible(S), and intermediate(I)**

|  | Susceptibility disk | S (μg/mL) | I (μg/mL) | R (μg/mL) |
| --- | --- | --- | --- | --- |
| 1 | Ampicillin | ≤ 8 | 16 | ≥ 32 |
| 2 | Cefazolin | ≤ 16 | - | ≥ 32 |
| 3 | Cefuroxime | ≤ 4 | 8-16 | ≥ 32 |
| 4 | Cefotaxime | ≤ 1 | 2 | ≥ 4 |
| 5 | Ceftazidime | ≤ 4 | 8 | ≥ 16 |
| 6 | Cefepime | ≤ 2 | 4-8 | ≥ 16 |
| 7 | Gentamicin | ≤ 2 | 4 | ≥ 8 |
| 8 | Tetracycline | ≤ 4 | 8 | ≥ 16 |
| 9 | Aztreonam | ≤ 4 | 8 | ≥ 16 |
| 10 | Cefoxitin | ≤ 8 | 16 | ≥ 32 |
| 11 | Imipenem | ≤ 1 | 2 | ≥ 4 |
| 12 | Meropenem | ≤ 1 | 2 | ≥ 4 |
| 13 | Amikacin | ≤ 4 | 8 | ≥ 16 |
| 14 | Levofloxacin | ≤ 0.5 | 1 | ≥ 2 |
| 15 | Tigecycline | ≤ 2 | 4 | ≥ 8 |
| 16 | Compound-Sulfamethoxazole | ≤ 2/38 | - | ≥ 4/76 |
| 17 | Cefoperazone-Sulbactam (2:1) | ≤ 16 | 32 | ≥ 64 |
| 18 | Ampicillin-Sulbactam | ≤ 8/4 | 16/8 | ≥32/16 |
| 19 | Ceftazidime-Avibactam | ≤ 8/4 | - | ≥16/4 |
| 20 | Piperacillin-Tazobactam | ≤ 8/4 | - | ≥32/4 |
| 21 | Amoxicillin-clavulanate | ≤ 8/4 | 16/8 | ≥32/16 |
