# Supplementary material for: Genomic and Pathogenic Characterization of an Extensively Drug‐Resistant Avian Pathogenic Escherichia coli Strain
Source: Transbound Emerg Dis. 2026 May 14;2026:4233989. doi: 10.1155/tbed/4233989 (PMC13173369; doi:10.1155/tbed/4233989)
Supplement: Supplementary file 3 — Supporting Information 3 Figure S1: Growth curve of isolated strains. Growth curve of the APEC strain SDLYRA415 was determined by measuring the optical density at 600 nm (OD600) every 30 min for 24 h at 37°C in LB medium. The growth curve showed that SDLYRA415 exhibited a lag phase from 0 to 1 h, a logarithmic phase from 1.5 to 15 h, and a stationary phase from 15.5 to 24 h, indicating normal growth activity of the strain under laboratory conditions. Figure S2: Genome map of the SDLYRA415 chromosome and plasmids. (A) Circular genome map of the SDLYRA415 chromosome, showing the distribution and orientation of predicted coding sequences, COG functional annotation, GC content, and GC skew. The complete genome of SDLYRA415 is 5.51 Mb with a GC content of 50.61%, consisting of one chromosome and four plasmids. (B) Circular genome map of plasmid pSDLYRA415‐1, which belongs to IncHI2/IncHI2A/IncN replicon type. The map presents predicted coding sequences, GC content, GC skew and the location of key resistance genes including MCR-1 and bla CTX-M-55. (C) Circular genome map of plasmid pSDLYRA415‐2, which belongs to IncFIB(AP001918)/IncFIC(FII) replicon type. The map displays predicted coding sequences, GC content, GC skew, and the distribution of virulence‐related genes. (D) Circular genome map of plasmid pSDLYRA415‐3, which is identified as a novel plasmid type with no known matched replicon. The map shows predicted coding sequences, GC content, GC skew, and the location of the carbapenem resistance gene blaNDM-5. (E) Circular genome map of plasmid pSDLYRA415‐4, which belongs to p0111 replicon type. The map illustrates predicted coding sequences, GC content and GC skew. Figure S3: PCR identification results of conjugated plasmid. Agarose gel electrophoresis was used to verify the transfer of four plasmids from donor strain SDLYRA415 to recipient strain EC600. M: DL5000 DNA marker (5000, 3000, 2000, 1500, 1000, 750, 500, 250, and 100 bp). 1: PCR product of pSDLYRA415‐1 from [file TBED-2026-4233989-s003.pdf]

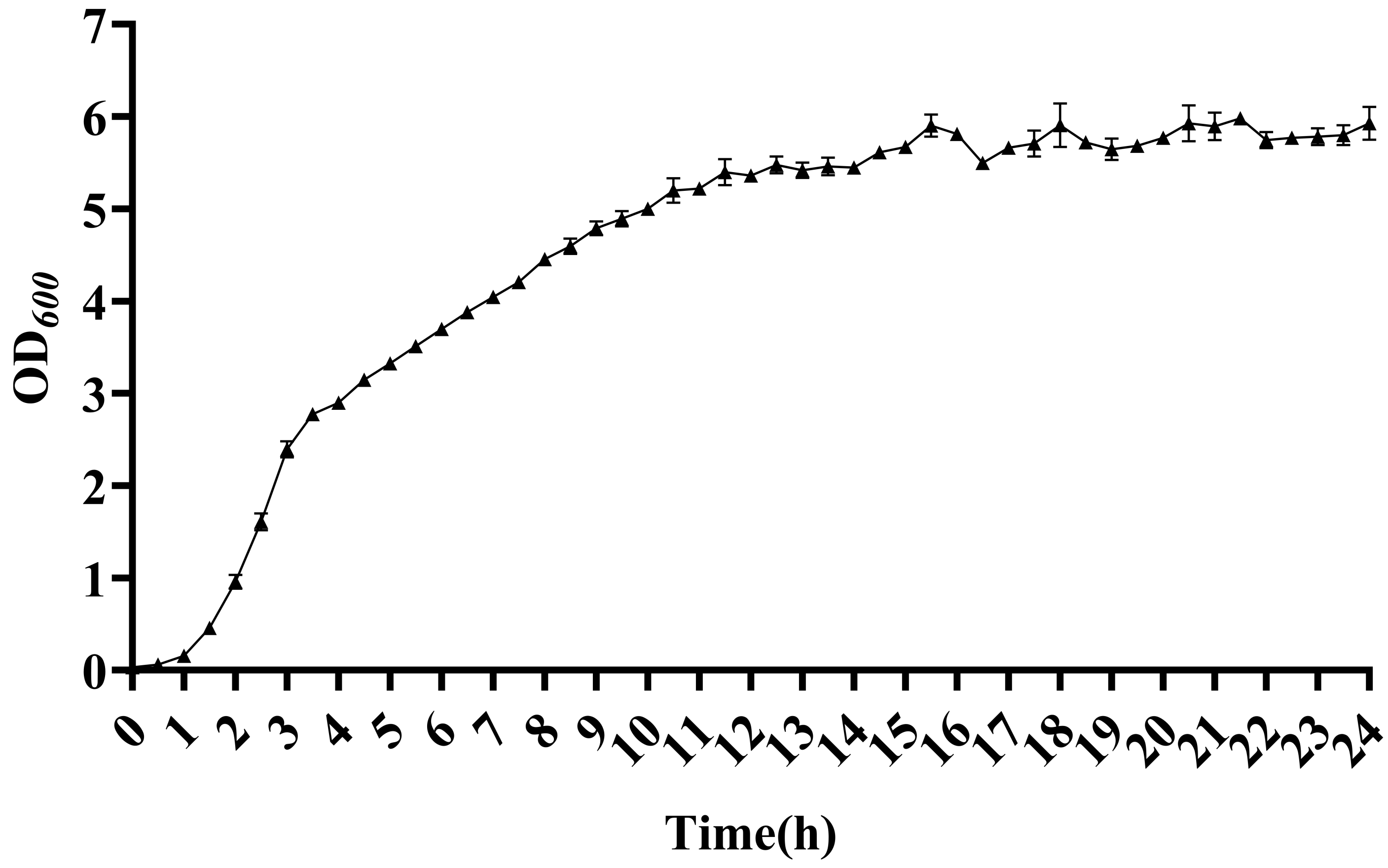

OD600
0
1
2
3
4
5
6
7
0 1 2 3 4 5 6 7 8 9 10 11 12 13 14 15 16 17 18 19 20 21 22 23 24
Time(h)

Figure S1

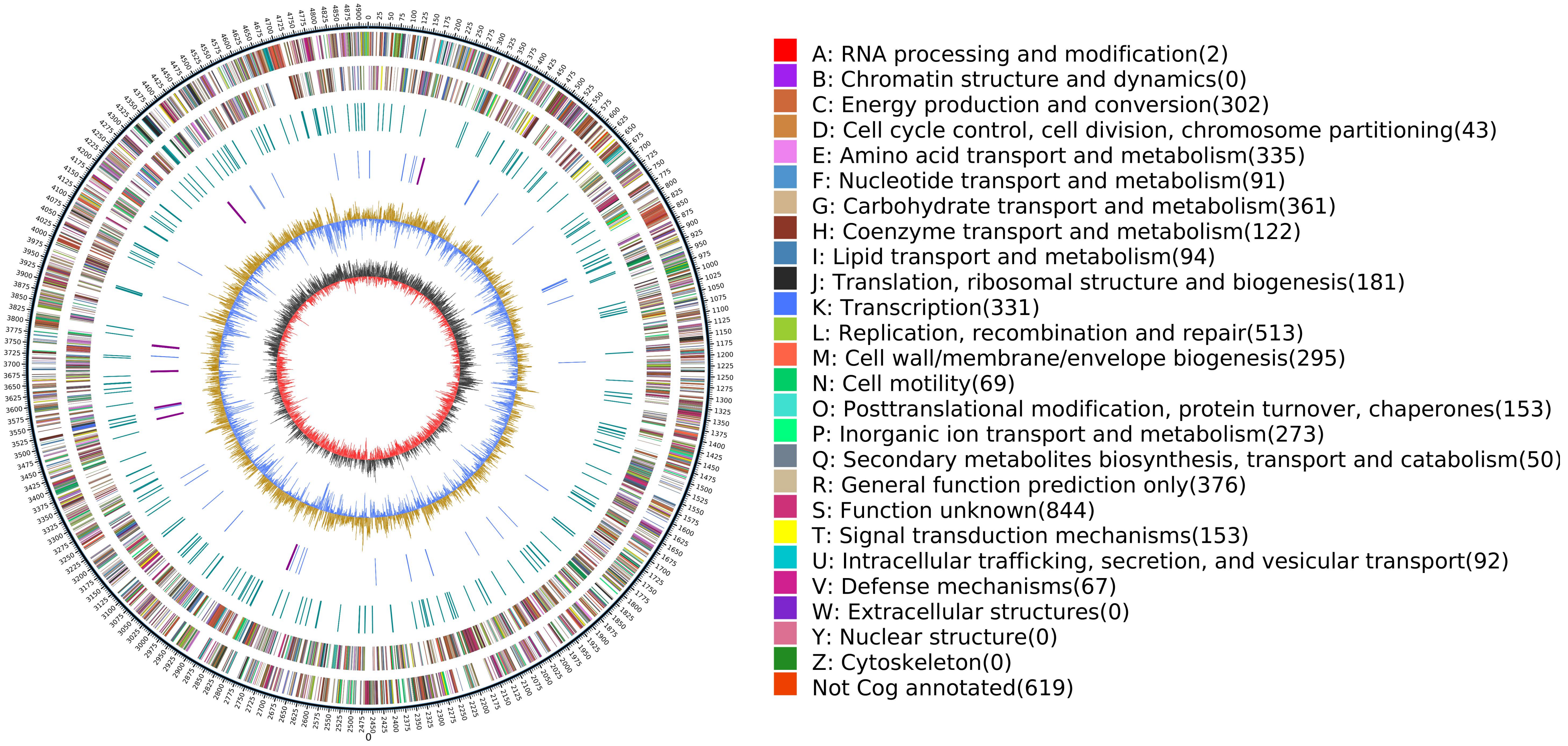
A: RNA processing and modification(2)
B: Chromatin structure and dynamics(0)
C: Energy production and conversion(302)
D: Cell cycle control, cell division, chromosome partitioning(43)
E: Amino acid transport and metabolism(335)
F: Nucleotide transport and metabolism(91)
G: Carbohydrate transport and metabolism(361)
H: Coenzyme transport and metabolism(122)
I: Lipid transport and metabolism(94)
J: Translation, ribosomal structure and biogenesis(181)
K: Transcription(331)
L: Replication, recombination and repair(513)
M: Cell wall/membrane/envelope biogenesis(295)
N: Cell motility(69)
O: Posttranslational modification, protein turnover, chaperones(153)
P: Inorganic ion transport and metabolism(273)
Q: Secondary metabolites biosynthesis, transport and catabolism(50)
R: General function prediction only(376)
S: Function unknown(844)
T: Signal transduction mechanisms(153)
U: Intracellular trafficking, secretion, and vesicular transport(92)
V: Defense mechanisms(67)
W: Extracellular structures(0)
Y: Nuclear structure(0)
Z: Cytoskeleton(0)
Not Cog annotated(619)

Figure S2A

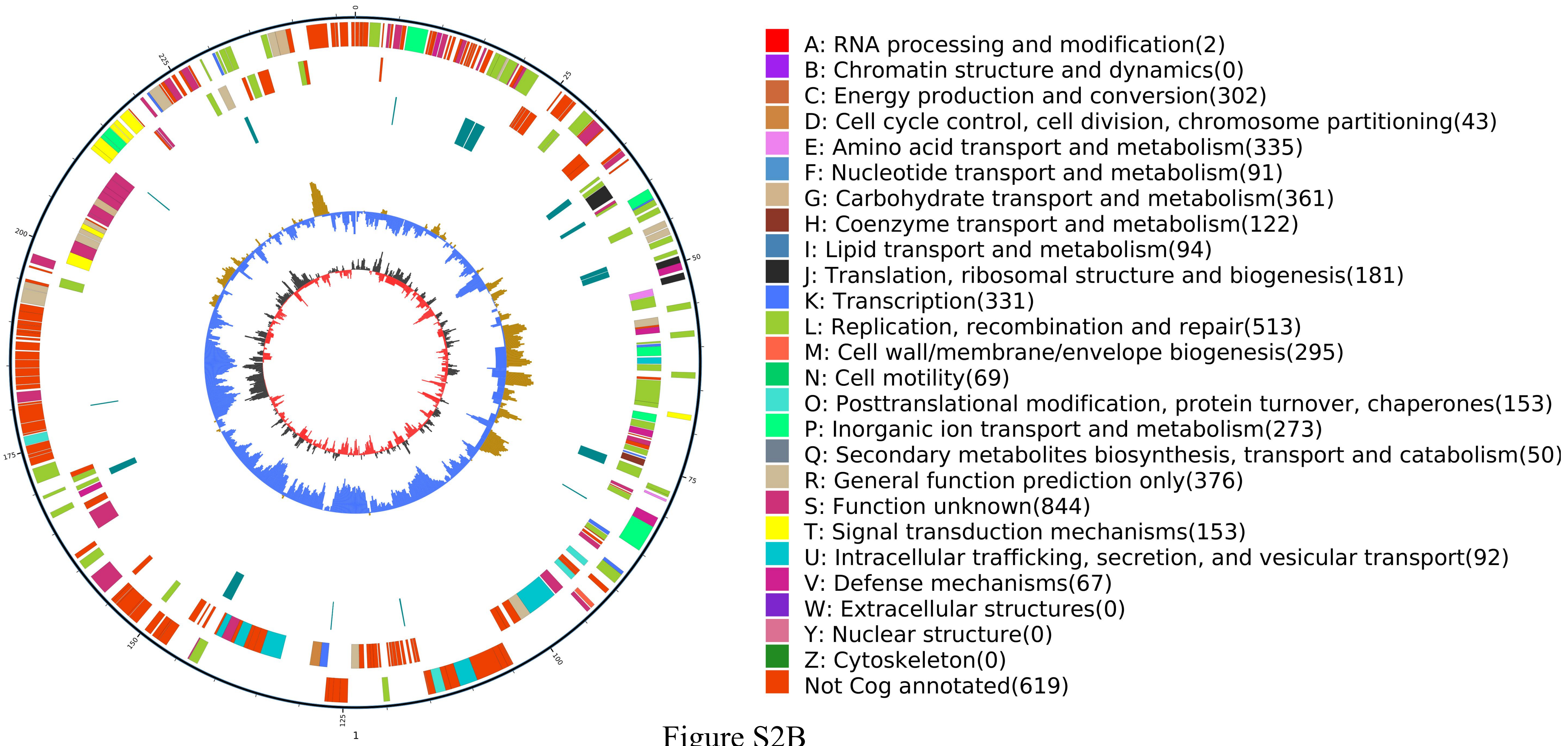
0
25
50
75
100
125
150
175
200
225
1
A: RNA processing and modification(2)
B: Chromatin structure and dynamics(0)
C: Energy production and conversion(302)
D: Cell cycle control, cell division, chromosome partitioning(43)
E: Amino acid transport and metabolism(335)
F: Nucleotide transport and metabolism(91)
G: Carbohydrate transport and metabolism(361)
H: Coenzyme transport and metabolism(122)
I: Lipid transport and metabolism(94)
J: Translation, ribosomal structure and biogenesis(181)
K: Transcription(331)
L: Replication, recombination and repair(513)
M: Cell wall/membrane/envelope biogenesis(295)
N: Cell motility(69)
O: Posttranslational modification, protein turnover, chaperones(153)
P: Inorganic ion transport and metabolism(273)
Q: Secondary metabolites biosynthesis, transport and catabolism(50)
R: General function prediction only(376)
S: Function unknown(844)
T: Signal transduction mechanisms(153)
U: Intracellular trafficking, secretion, and vesicular transport(92)
V: Defense mechanisms(67)
W: Extracellular structures(0)
Y: Nuclear structure(0)
Z: Cytoskeleton(0)
Not Cog annotated(619)

Figure S2B

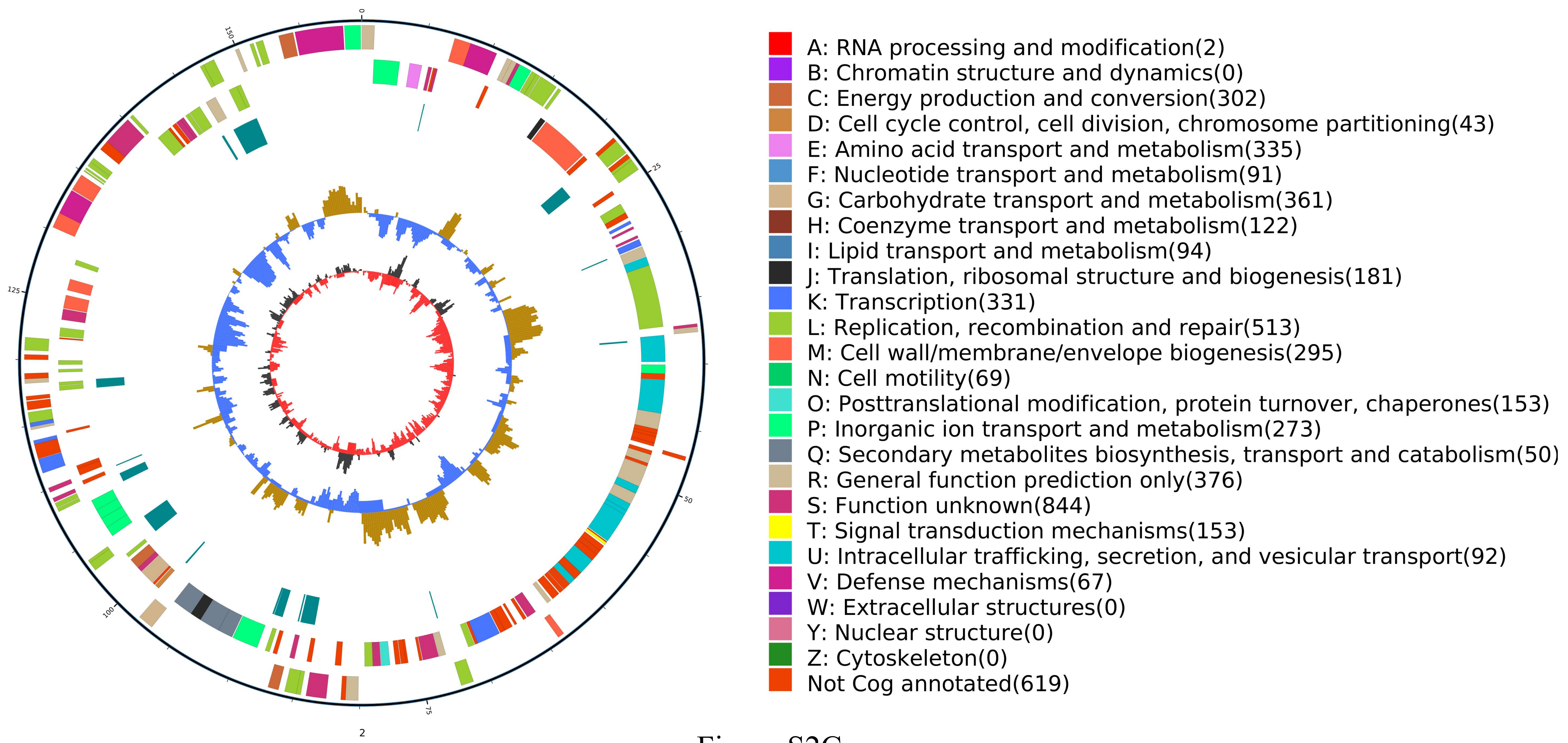
0
25
50
75
100
125
150
2
A: RNA processing and modification(2)
B: Chromatin structure and dynamics(0)
C: Energy production and conversion(302)
D: Cell cycle control, cell division, chromosome partitioning(43)
E: Amino acid transport and metabolism(335)
F: Nucleotide transport and metabolism(91)
G: Carbohydrate transport and metabolism(361)
H: Coenzyme transport and metabolism(122)
I: Lipid transport and metabolism(94)
J: Translation, ribosomal structure and biogenesis(181)
K: Transcription(331)
L: Replication, recombination and repair(513)
M: Cell wall/membrane/envelope biogenesis(295)
N: Cell motility(69)
O: Posttranslational modification, protein turnover, chaperones(153)
P: Inorganic ion transport and metabolism(273)
Q: Secondary metabolites biosynthesis, transport and catabolism(50)
R: General function prediction only(376)
S: Function unknown(844)
T: Signal transduction mechanisms(153)
U: Intracellular trafficking, secretion, and vesicular transport(92)
V: Defense mechanisms(67)
W: Extracellular structures(0)
Y: Nuclear structure(0)
Z: Cytoskeleton(0)
Not Cog annotated(619)

Figure S2C

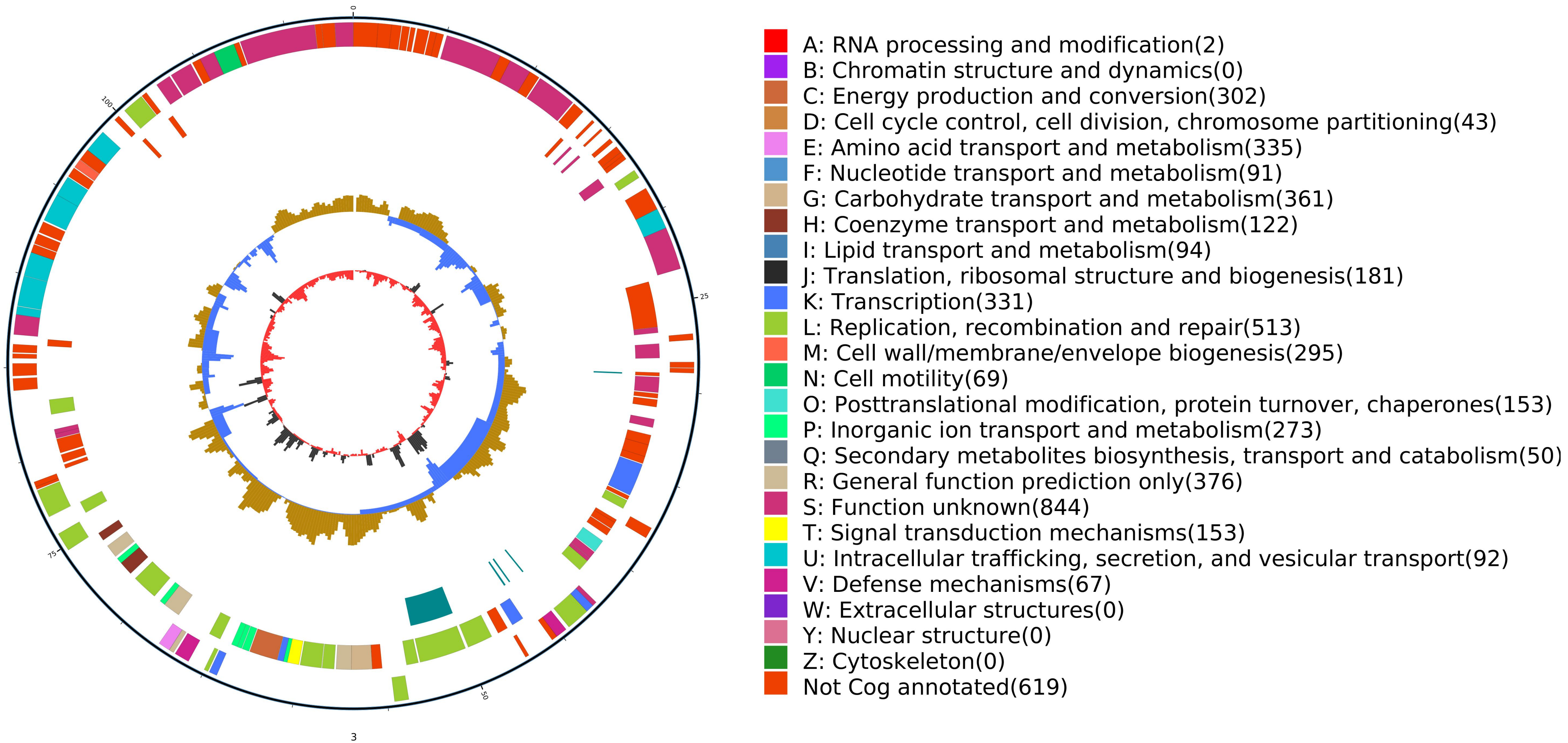
0
25
50
75
100
3
A: RNA processing and modification(2)
B: Chromatin structure and dynamics(0)
C: Energy production and conversion(302)
D: Cell cycle control, cell division, chromosome partitioning(43)
E: Amino acid transport and metabolism(335)
F: Nucleotide transport and metabolism(91)
G: Carbohydrate transport and metabolism(361)
H: Coenzyme transport and metabolism(122)
I: Lipid transport and metabolism(94)
J: Translation, ribosomal structure and biogenesis(181)
K: Transcription(331)
L: Replication, recombination and repair(513)
M: Cell wall/membrane/envelope biogenesis(295)
N: Cell motility(69)
O: Posttranslational modification, protein turnover, chaperones(153)
P: Inorganic ion transport and metabolism(273)
Q: Secondary metabolites biosynthesis, transport and catabolism(50)
R: General function prediction only(376)
S: Function unknown(844)
T: Signal transduction mechanisms(153)
U: Intracellular trafficking, secretion, and vesicular transport(92)
V: Defense mechanisms(67)
W: Extracellular structures(0)
Y: Nuclear structure(0)
Z: Cytoskeleton(0)
Not Cog annotated(619)

Figure S2D

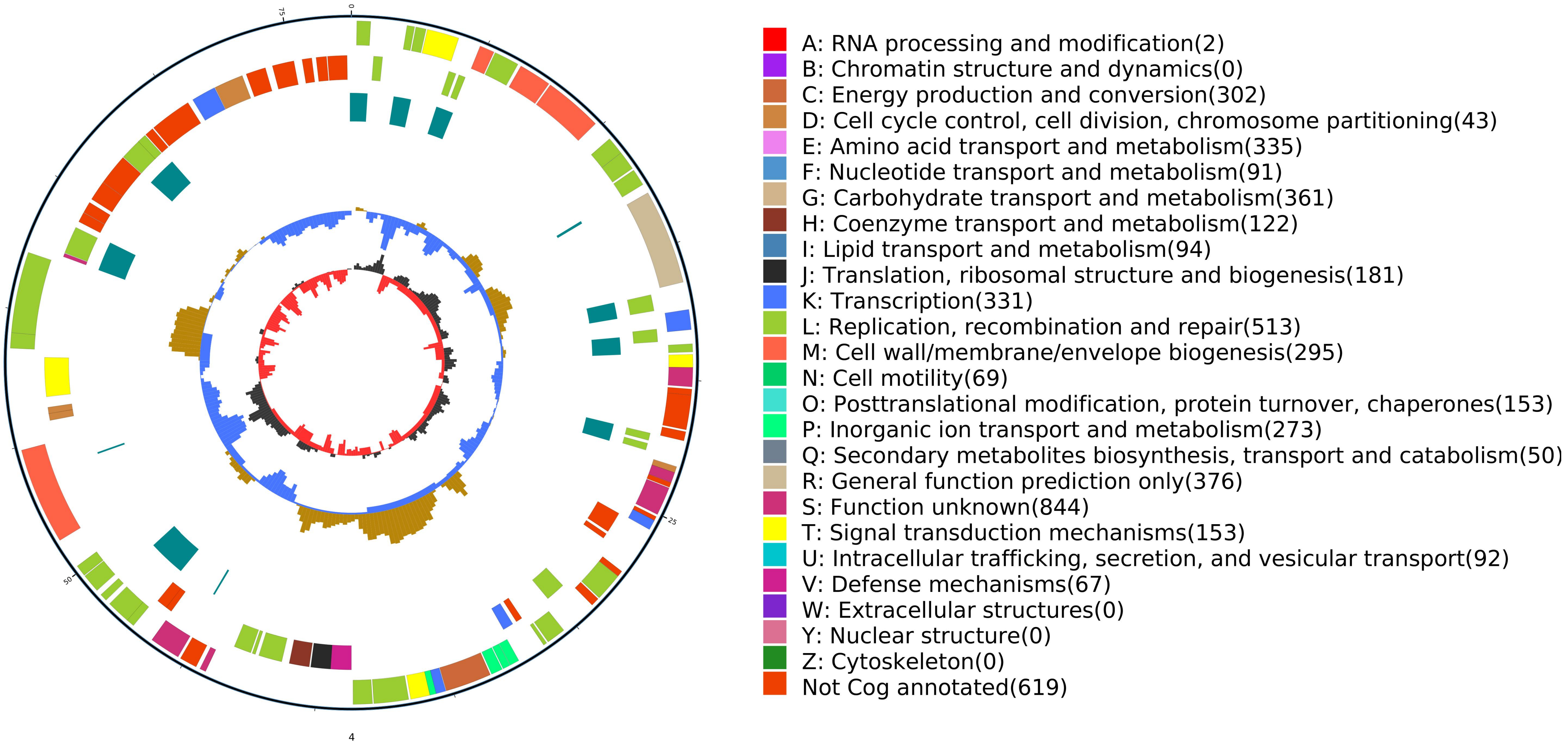
0
75
25
50
4
A: RNA processing and modification(2)
B: Chromatin structure and dynamics(0)
C: Energy production and conversion(302)
D: Cell cycle control, cell division, chromosome partitioning(43)
E: Amino acid transport and metabolism(335)
F: Nucleotide transport and metabolism(91)
G: Carbohydrate transport and metabolism(361)
H: Coenzyme transport and metabolism(122)
I: Lipid transport and metabolism(94)
J: Translation, ribosomal structure and biogenesis(181)
K: Transcription(331)
L: Replication, recombination and repair(513)
M: Cell wall/membrane/envelope biogenesis(295)
N: Cell motility(69)
O: Posttranslational modification, protein turnover, chaperones(153)
P: Inorganic ion transport and metabolism(273)
Q: Secondary metabolites biosynthesis, transport and catabolism(50)
R: General function prediction only(376)
S: Function unknown(844)
T: Signal transduction mechanisms(153)
U: Intracellular trafficking, secretion, and vesicular transport(92)
V: Defense mechanisms(67)
W: Extracellular structures(0)
Y: Nuclear structure(0)
Z: Cytoskeleton(0)
Not Cog annotated(619)

Figure S2E

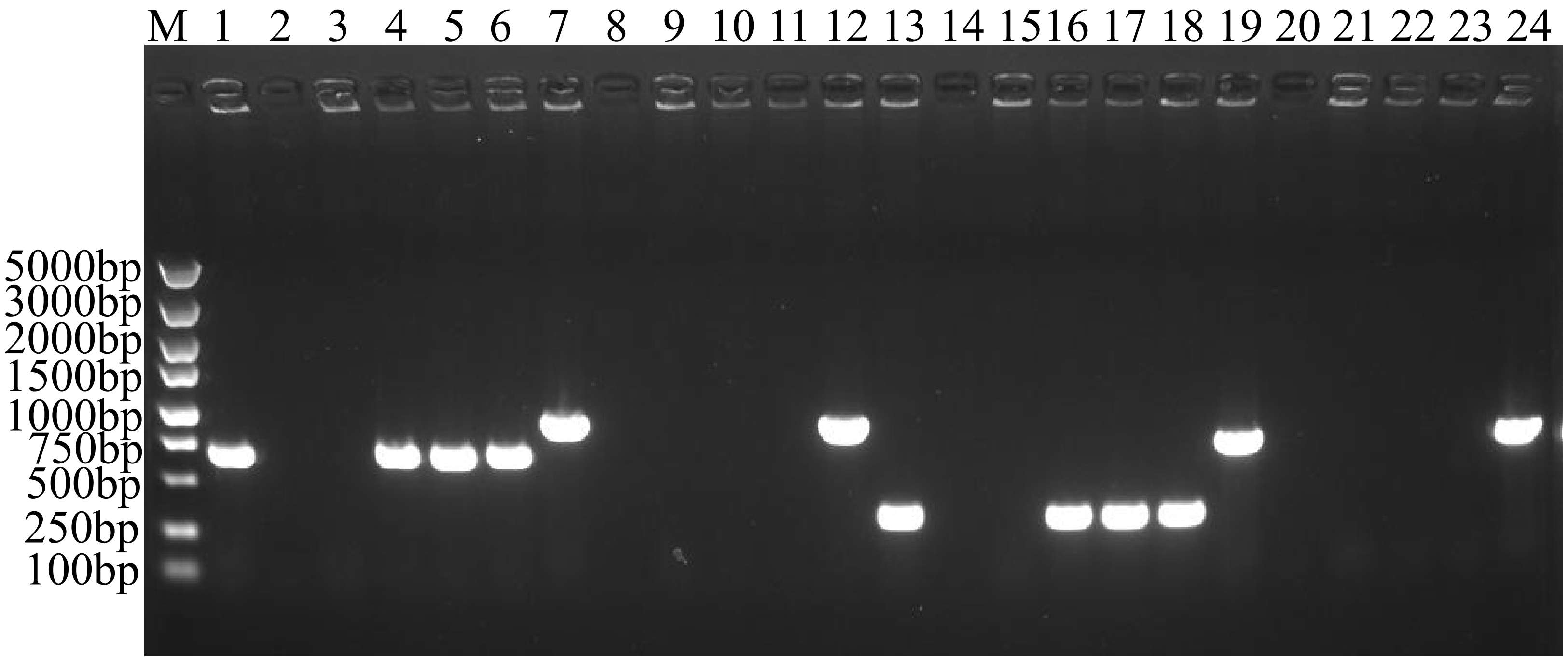
M 1 2 3 4 5 6 7 8 9 10 11 12 13 14 15 16 17 18 19 20 21 22 23 24
5000bp
3000bp
2000bp
1500bp
1000bp
750bp
500bp
250bp
100bp

Figure S3
